# A New Hybrid Inversion Method for 2D Nuclear Magnetic Resonance Combining TSVD and Tikhonov Regularization

**DOI:** 10.3390/jimaging7020018

**Published:** 2021-01-28

**Authors:** Germana Landi, Fabiana Zama, Villiam Bortolotti

**Affiliations:** 1 Department of Mathematics, University of Bologna, 40126 Bologna, Italy; germana.landi@unibo.it; 2 Department of Civil, Chemical, Environmental, and Materials Engineering, University of Bologna, 40126 Bologna, Italy; villiam.bortolotti@unibo.it

**Keywords:** hybrid regularization method, truncated singular values decomposition, tikhonov method, Nuclear Magnetic Resonance (NMR) relaxometry

## Abstract

This paper is concerned with the reconstruction of relaxation time distributions in Nuclear Magnetic Resonance (NMR) relaxometry. This is a large-scale and ill-posed inverse problem with many potential applications in biology, medicine, chemistry, and other disciplines. However, the large amount of data and the consequently long inversion times, together with the high sensitivity of the solution to the value of the regularization parameter, still represent a major issue in the applicability of the NMR relaxometry. We present a method for two-dimensional data inversion (2DNMR) which combines Truncated Singular Value Decomposition and Tikhonov regularization in order to accelerate the inversion time and to reduce the sensitivity to the value of the regularization parameter. The Discrete Picard condition is used to jointly select the SVD truncation and Tikhonov regularization parameters. We evaluate the performance of the proposed method on both simulated and real NMR measurements.

## 1. Introduction

Nuclear Magnetic Resonance (NMR) relaxometry has become an important tool to study the molecular structure and properties of materials. A typical NMR experiment consists of measuring the relaxation process due to the re-establishment of the nuclear system into its equilibrium state, after the application of a short magnetic pulse parameterized with a predefined flip angle. The relaxation process is described by longitudinal and transversal dynamics, characterized by distributions of longitudinal ($T_{1}$) and transversal ($T_{2}$) relaxation times [1]. The computation of the relaxation times distribution requires the numerical solution of a Fredholm integral equation with separable Laplace-type kernels. In particular, we focus on the inversion of 2D NMR relaxation data, acquired using a conventional Inversion-Recovery (IR) experiment detected by a Carr-Purcell-Meiboom-Gill (CPMG) pulse train [2]. Then, the evolution time $t_{1}$ in IR and the evolution time $t_{2}$ in CPMG are two independent variables, and the 2D NMR relaxation data $S(t_{1},t_{2})$ can be expressed as:

(1) $$S\left(t_{1},t_{2}\right)=\overset{\;\infty }{\underset{0}{\;\int \int \;}}k_{1}\left(t_{1},T_{1}\right)k_{2}\left(t_{2},T_{2}\right)F\left(T_{1},T_{2}\right)\;dT_{1}\;dT_{2}+e\left(t_{1},t_{2}\right)$$

where the unknown $F(T_{1},T_{2})$ is the distribution of $T_{1}$ and $T_{2}$ relaxation times, $e(t_{1},t_{2})$ represents Gaussian additive noise and $k_{1}\left(t_{1},T_{1}\right)$, $k_{2}\left(t_{2},T_{2}\right)$ are Laplace-type kernels given by:

(2) $$k_{1}\left(t_{1},T_{1}\right)=1-2\exp\left(-t_{1}/T_{1}\right),\;k_{2}\left(t_{2},T_{2}\right)=\exp\left(-t_{2}/T_{2}\right)$$

whose singular values quickly decay to zero. Since the unknown function *F* corresponds to distribution of the values of the relaxation times $T_{1}-T_{2}$, we can assume $F(T_{1},T_{2})\geq 0$ for all $T_{1}$ and $T_{2}$. Experimental data is usually collected at discrete values of times; therefore by considering $M_{1}\times M_{2}$ samples of the times $t_{1}$ and $t_{2}$, and $N_{1}\times N_{2}$ samples of the relaxation times $T_{1}$ and $T_{2}$, problem (1) is discretized as:

(3) $$Kf+e=s,\;K=K_{2}⊗K_{1}$$

where $K_{1}\in R^{M_{1}\times N_{1}}$, $K_{2}\in R^{M_{2}\times N_{2}}$ are the discretized exponential kernels, $s\in R^{M}$, $M=M_{1}\cdot M_{2}$, is the discrete vector of the measured noisy data (which can have negative values), $f\in R^{N}$, $N=N_{1}\cdot N_{2}$, is the vector reordering of the unknown distribution and $e\in R^{M}$ is the vector with the discretized noise.

The inversion of (3) is severely ill-conditioned and several direct and iterative methods have been deeply discussed both in NMR [3,4,5] and mathematical [6,7,8] literature. Direct regularization includes Truncated Singular Value Decomposition (TSVD) and Tikhonov methods. TSVD replaces K by a matrix of reduced rank thus avoiding noise amplification in the inversion procedure. This is usually obtained by removing the singular values smaller than a given tolerance τ>0. Tikhonov-like regularization substitutes (3) with a more stable optimization problem whose objective function includes a data-fitting term and a regularization term imposing some *a priori* knowledge about the solution. In NMR, Tikhonov-like regularization is usually employed. It requires the solution of a non-negative least squares (NNLS) problem of the form

(4) $$\min_{f\geq 0}\;\left\{{∥Kf-s∥}^{2}+\alpha {∥f∥}^{2}\right\}$$

where α is the regularization parameter. In the sequel, ∥·∥ will denote the Euclidean norm of a vector or the Frobenius norm of a matrix. It is well-known that α controls the smoothness in f and makes the inversion less ill-conditioned but, if wrongly chosen, it may cause a bias to the result. For this reason, many parameter selection techniques have been proposed such as Generalized Cross Validation (GCV), the L-curve and U-curve methods and methods based on the Discrepancy Principle (DP) (see [6] and references therein). In NMR, the Butler, Reed, and Dawson (BRD) method [9] or the S-curve method [10] are usually applied for the selection of the regularization parameter.

Another critical issue when solving NMR problems is that the amount of data collected for one measurement can be very large, with typical sizes $M_{1}=30$–150 and $M_{2}=1000$–50,000. If a grid used for the estimated distribution has 100×100 elements, then the matrix K can have up to $5\cdot 10^{9}$ elements and the computational cost of the data inversion may be extremely high.

The separable kernel structure of K has been widely exploited in the NMR literature to perform data compression using independent SVDs of $K_{1}$ and $K_{2}$ and reduce computational complexity. The method proposed in [4], in the sequel referred to as Venkataramanan–Song–Hürlimann (VSH) algorithm, is considered a reference method for NMR data inversion and it is widely used both in academia and industrial world. Given the TSVD of $K_{1}$ and $K_{2}$:

(5) $$K_{k_{i},i}={\bar{U}}_{k_{i},i}{\bar{\Sigma }}_{k_{i},i}{\bar{V}}_{k_{i},i}^{T},\;i=1,2$$

where $k_{i}$ is the number of considered singular values, the VSH method approximates the original problem (4) with the better conditioned one

(6) $$\min_{f\geq 0}\left\{∥K_{k_{1},k_{2}}{f-s∥}^{2}+\alpha {∥f∥}^{2}\right\}$$

where $K_{k_{1},k_{2}}=K_{k_{2},2}⊗K_{k_{1},1}$. The data s is then projected onto the column subspace of $K_{k_{1},k_{2}}$ in order to obtain a NNLS problem with compressed data and kernel of significantly smaller dimensions. A method adapted from the BRD algorithm is then used to solve the optimization problem.

Other methods have been proposed in the literature for the inversion of 2DNMR data. The algorithm of Chouzenoux et al. [11] uses a primal-dual optimization strategy coupled with an iterative minimization in order to jointly account for the non-negativity constraint in (4) and introduce a regularization term. A preconditioning strategy is used to reduce the computational cost of the algorithm. In [12], an algorithm for 2DNMR inversion from limited data is presented. Such algorithm uses compressive sensing-like techniques to fill in missing measurements and the resulting regularized minimization problem is solved using the method of [4]. The Discrete Picard Condition (DPC) [13] is used to choose the truncation parameters. The 2DUPEN algorithm [14] uses multiparameter Tikhonov regularization with automatic choice of the regularization parameters. Without any a-priori information about the noise norm, 2DUPEN automatically computes the locally adapted regularization parameters and the distribution of the unknown NMR parameters by using variable smoothing. In [15], an improvement of 2DUPEN (I2DUPEN), obtained by applying data windowing and SVD filtering, is presented. The SVD filtering is applied to reduce the problem size as in [4].

Since the matrices $K_{1}$ and $K_{2}$ have typically small sizes, it is possible to compute the exact SVD of K by exploiting its Kronecker product structure. In fact, if $K=U\Sigma V^{T}$ is the SVD of K, then

(7) $$U={\bar{U}}_{2}⊗{\bar{U}}_{1},\;\Sigma ={\bar{\Sigma }}_{2}⊗{\bar{\Sigma }}_{1},\;V={\bar{V}}_{2}⊗{\bar{V}}_{1}.$$

However, the TSVD of K cannot be computed as a Kronecker product. For this reason, different approximation of the TSVD can be found in the literature such as the matrix $K_{k_{1},k_{2}}$ of the VSH method or the randomized SVD (RSVD) [16,17] where randomized algorithms are used to approximate the dominant singular values of K. In this work, we show that it is possible to compute the exact TSVD of K efficiently, avoiding approximations and suitably using properties of the Kronecker product structure. Let $K_{k}=U_{k}\Sigma_{k}V_{k}^{T}$ be the TSVD of K where *k* is the number of considered singular values; we propose to solve the following Tikhonov-like problem:

(8) $$\min_{f\geq 0}\;\left\{∥K_{k}{f-s∥}^{2}+\alpha {∥f∥}^{2}\right\}.$$

Moreover, we propose and test an automatic rule, based on the DPC, for the automatic selection of both the TSVD truncation index and the Tikhonov parameter. Finally, we analyze the filtering properties of our method compared to TSVD, Tikhonov and VSH methods. Therefore, our approach can be consideredt o be a hybrid inversion method that combines TSVD and Tikhonov regularization: Tikhonov regularization prevents from discontinuities and artificial peaks and TSVD acts as a preconditioning strategy and reduces the computational cost. Actually, other approaches do exist in the literature combining RTSVD and Tikhonov regularization [16,17]. Such techniques apply randomized algorithms to reduce large-scale problems to much smaller-scale ones and find a regularized solution by applying some regularization method to the small-scale problems in combination with some parameter choice rule. The key point of these randomized algorithms is that the SVD of the original linear operator K is never computed.

Concerning the solution of (8), in the present paper we apply the Newton Projection (NP) method [18] where the Conjugate Gradient (CG) method is used to solve the inner linear systems. This technique guarantees the desired high accuracy and it has been successfully applied in the NMR context [14,15]. Gradient projection methods with acceleration techniques such those discussed in [19] required more computational effort to solve (8) with the required accuracy. However, the typical sparse structure of the solution, represented by nonzero peaks over flat regions, could possibly be taken into account in solving (8), for example by adding L1 penalties [20].

Summarizing, the contribution of this paper is twofold; first, the paper shows that times distribution of improved quality can be obtained by using $K_{k}$ instead of the separate TSVDs of $K_{1}$ and $K_{2}$ without a significant increase in the computational complexity. In fact, the computational cost of our approach is slightly greater than the cost of VSH and considerably smaller than the cost of RSVD. Second, the paper describes an efficient method for jointly selecting the SVD truncation index *k* and the Tikhonov regularization parameter α and for solving the NMR data inversion problem.

The remainder of this paper is organized as follows. In Section 2 we introduce our method and, in Section 3, we analyze its filtering properties. In Section 4, we provide implementation details and discuss numerical results. Some conclusions are reported in Section 5. Finally, the VSH method is described in Appendix C.

## 2. The Proposed Hybrid Algorithm

In this section we illustrate the details of our approach and formalize our proposed Hybrid Algorithm 1. We first propose to approximate problem (4) with

(9) $$\min_{f\geq 0}\;\left\{∥K_{k}{f-s∥}^{2}+\alpha {∥f∥}^{2}\right\}.$$

where $K_{k}=U_{k}\Sigma_{k}V_{k}^{T}$ is the TSVD of K. By projecting the data vector s onto the column subspace of $K_{k}$ and by neglecting constant terms, we obtain the equivalent formulation:

(10) $$\min_{f\geq 0}\;\left\{∥U_{k}U_{k}^{T}K_{k}f-U_{k}U_{k}^{T}{s∥}^{2}+\alpha {∥f∥}^{2}\right\}$$

which can be written as:

(11) $$\min_{f\geq 0}\;\left\{∥\Sigma_{k}V_{k}^{T}f-U_{k}^{T}{s∥}^{2}+\alpha {∥f∥}^{2}\right\}$$

with compressed data $U_{k}^{T}s\in R^{k}$ and kernel $\Sigma_{k}V_{k}^{T}$. We observe that the solution of (11) lies in a subspace of the column space of $V_{k}$. In the following paragraphs we develop the solution steps of problem (11), the rule for the choice of the TSVD truncation index *k*, and of the Tikhonov parameter α. Finally, we illustrate the filtering properties of our new method and compare it to Tikhonov, TSVD and VSH methods.

### 2.1. Solution of the Minimization Problem

We use the Newton Projection (NP) method [18] to solve the constrained minimization problem (11); for a detailed description of NP we refer the reader to Appendix B. We apply the Conjugate Gradient (CG) method to solve the inner linear systems. To this purpose, it is necessary to perform several matrix-vector products involving the matrices $U_{k}$ and $V_{k}$. Although $U_{k}$ and $V_{k}$ are parts of the matrices U and V, they do not inherit the Kronecker product structure. However, we can show that matrix-vector products can be performed efficiently by exploiting the structure of U and V.

Given a vector $x\in R^{M}$, let zero(x,k) be the vector obtained by zeroing the last M−k components of x:

(12) $$\mathrm{zero}\left(x,k\right)={\left(x_{1},x_{2},\ldots ,x_{k},0,\ldots ,0\right)}^{T}.$$

Using the Kronecker products property:

(13) $$\left(A⊗B\right)\mathrm{vec}\left(X\right)=\mathrm{vec}\left({\mathrm{BXA}}^{T}\right).$$

From Equation (7) we have

(14) $$U_{k}^{T}s=\mathrm{zero}\left(U^{T}s,k\right)=\mathrm{zero}\left(\mathrm{vec}\left({\bar{U}}_{1}^{T}S{\bar{U}}_{2}\right),k\right)$$

and

(15) $$V_{k}^{T}f=\mathrm{zero}\left(V^{T}f,k\right)=\mathrm{zero}\left(\mathrm{vec}\left({\bar{V}}_{1}^{T}F{\bar{V}}_{2}\right),k\right).$$

where $S\in R^{M_{1}\times M_{2}}$ is the matrix of the measured data s.t. s=vec(S) and $F\in R^{N_{1}\times N_{2}}$ represents the computed distribution s.t. f=vec(F). Thus, matrix-vector products involving $U_{k}$ and $V_{k}$ can be efficiently performed by using the Kronecker product structure of U and V and by setting to zero the last components of the resulting vector.

### 2.2. Choice of the Parameters *k* and α

The quality of the restored distribution f strongly depends on the values of both the truncation parameter *k* and the Tikhonov parameter α. We propose to choose both these values by using the Discrete Picard Condition (DPC) [6]. This condition, for ill-conditioned inverse problems, guarantees that a good solution is obtained by keeping in the SVD expansion for the solution $f=\sum_{i}\frac{u_{i}^{T}s}{\sigma_{i}}v_{i}$ only the coefficients $u_{i}^{T}s/\sigma_{i}$ such that the Fourier coefficients $u_{i}^{T}s$ decrease on average faster than the singular values $\sigma_{i}$. A truncation parameter *k* satisfying the DPC can be selected by visual inspection of the so-called Picard plot (i.e., a plot of the quantities $u_{i}^{T}s$ and $\sigma_{i}$ versus *i*). Alternatively, an index *k* satisfying the DPC can also be selected by using automatic techniques such as those described in [21,22].

Once the value for *k* has been fixed by using the DPC, the value for α is set as

(16) $$\alpha =\sigma_{k}^{2}$$

since, as explained in [6], the value $\alpha =\sigma_{i}^{2}$ represents the breakpoint at which the *i*th filter factor changes nature for the Tikhonov method [6]. Therefore this choice is motivated by the low-pass filtering properties of both TSVD and Tikhonov methods.

Summarizing the previous considerations, we outline the steps of our proposed Hybrid Algorithm 1.
**Algorithm 1:** Hybrid algorithm1: compute the SVDs of $K_{1}$ and $K_{2}$2: compute $\Sigma =\Sigma_{2}⊗\Sigma_{1}$3: choose *k* by using the DPC4: choose $\alpha =\sigma_{k}^{2}$5: Apply the Newton projection method to solve the constrained problem
$$\min_{f\geq 0}\;∥\Sigma_{k}V_{k}^{T}f-U_{k}^{T}{s∥}^{2}+\alpha {∥f∥}^{2}$$

## 3. Analysis of the Filtering Properties

In this section we prove that our Hybrid algorithm acts as a low-pass filtering method, similarly to TSVD and Tikhonov methods, and we compare it to the filtering properties of VSH. Let us first report some basic properties of the solution of the NNLS problem:

(17) $$\min_{f\geq 0}\;{∥Kf-s∥}^{2}.$$

Assume that $f^{*}$ is a solution of (17), then it satisfies [23]:

(18) $${\left[K^{T}Kf^{*}-K^{T}s\right]}_{i}=0,\;\mathrm{for}\;\mathrm{all}\;i\;\mathrm{such}\;\mathrm{that}\;f_{i}^{*}>0.$$

Once defined the active set of $f^{*}$ by

(19) $$A^{*}=\left\{i\;|\;f_{i}^{*}=0\right\},$$

we can define the diagonal matrix $D^{*}$ as

(20) $${\left[D^{*}\right]}_{ii}=\left\{\begin{array}{cc} 1, & i\notin A^{*};\\ 0, & i\in A^{*}. \end{array}\right.$$

Then, using the relation $D^{*}f^{*}=f^{*}$, we obtain from (18)

(21) $$D^{*}K^{T}KD^{*}f^{*}-D^{*}K^{T}s=0$$

which are the normal equations of the following LS problem

(22) $$\min_{f}\;{∥KD^{*}f-s∥}^{2}.$$

The following result characterises the minimum norm solution of (22).

**Theorem 1.**  *Let*$K\in R^{N\times M}$, $s\in R^{N}$*and let*$D^{*}$*be the diagonal matrix defined as* (20) where $A^{*}$ *is the active set of a local solution of (17). Then, the minimum norm solution of the least squares problem (17) has the following expression*

(23) $$f^{*}=\sum_{i=1}^{N}\frac{u_{i}^{T}s}{\sigma_{i}}D^{*}v_{i}.$$

To prove this result, we first need the following lemma whose proof is given, for the reader’s convenience, in Appendix A.

**Lemma 1.**  *Let* $K=U\Sigma V^{T}$ *be the SVD of* $K\in R^{M\times N}$ *and let* $D^{*}\in R^{N\times N}$ *be the diagonal matrix defined in (20). Then, the pseudo-inverse of* $U\Sigma V^{T}D^{*}\in R^{M\times N}$ *is given by*

(24) $${\left(U\Sigma V^{T}D^{*}\right)}^{†}=D^{*}V\Sigma^{†}U^{T}.$$

We are now in the position to prove Theorem 1.

**Proof.**  (Theorem 1) Using the pseudo-inverse notation, we can write the solution of the LS problem (22) as:

$$f^{*}={\left(KD^{*}\right)}^{†}s={\left(U\Sigma V^{T}D^{*}\right)}^{†}s$$

and, using (24) we have:

$$f^{*}=D^{*}V\Sigma^{†}U^{T}s$$

hence

$$f^{*}=D^{*}\sum_{i=1}^{N}\frac{u_{i}^{T}s}{\sigma_{i}}v_{i}=\sum_{i=1}^{N}\frac{u_{i}^{T}s}{\sigma_{i}}D^{*}v_{i}.$$

□

Theorem 1 shows that the solution of the constrained problem (17) can be written in terms of the SVD of matrix K as follows:

$$f^{*}=\sum_{i=1}^{N}\frac{u_{i}^{T}s}{\sigma_{i}}{\hat{v}}_{i}\;\mathrm{where}\;{\hat{v}}_{i}=D^{*}v_{i}.$$

Obviously, the vectors ${\hat{v}}_{i}$ may be linear dependent if $f^{*}$ lies in a subspace of $R^{N}$. It is well known that TSVD and Tikhonov methods both compute a filtered solution $f_{\mathrm{filt}}$ of problem (22) with different filter functions $\phi_{i}\left(\cdot \right)$ [6]. Using the result of Theorem 1, we can express the nonnegatively constrained TSVD solution as

(25) $$f_{TSVD}=\sum_{i=1}^{N}\phi_{i}^{TSVD}\left(k\right)\frac{u_{i}^{T}s}{\sigma_{i}}{\hat{v}}_{i},\;{\hat{v}}_{i}=D^{*}v_{i}$$

where $D^{*}$ is the diagonal matrix (20) and the filter factors are

(26) $$\phi_{i}^{TSVD}\left(k\right)=\left\{\begin{array}{cc} 1, & \mathrm{if}\;i\leq k;\\ 0, & \mathrm{otherwise}; \end{array}\right.\;\mathrm{with}\;k\in \left\{1,\ldots ,N\right\}.$$

Analogously, the non negative Tikhonov solution is given by

(27) $$f_{TIKH}=\sum_{i=1}^{N}\phi_{i}^{TIKH}\left(\alpha \right)\frac{u_{i}^{T}s}{\sigma_{i}}{\hat{v}}_{i},\;{\hat{v}}_{i}=D_{TIKH}^{*}v_{i}$$

where $D_{TIKH}^{*}$ is the diagonal matrix (20) defined with respect to the active set $A^{*}$ of a local solution of (4) and the filter factors are

(28) $$\phi_{i}^{TIKH}\left(\alpha \right)=\frac{\sigma_{i}^{2}+\alpha }{\sigma_{i}^{2}},\;\mathrm{with}\;\alpha \in R^{+}.$$

**Figure 1:**
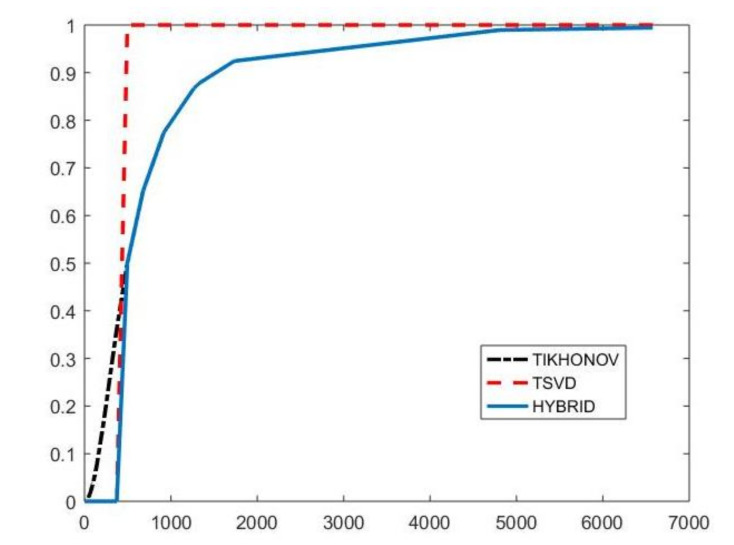
The Hybrid (blue line), TSVD (red dashed line) and Tikhonov (black dashdotted line) filter factors $\phi_{i}$ versus $\sigma_{i}$ for $\alpha =2.5\cdot 10^{5}$.

Equations (25) and (27) define a low-pass filter with vectors ${\hat{v}}_{i}$. The solution provided by the hybrid method is still a filtered solution whose filter factors depend on two parameters; i.e.,

(29) $$f_{HYBRID}=\sum_{i=1}^{N}\phi_{i}^{HYBRID}\left(\alpha ,k\right)\frac{u_{i}^{T}s}{\sigma_{i}}{\hat{v}}_{i},\;{\hat{v}}_{i}=D_{HYBRID}^{*}v_{i}$$

where $D_{HYBRID}^{*}$ is the diagonal matrix (20) defined with respect to the active set $A^{*}$ of a local solution of (11) and the filter factors are

(30) $$\phi_{i}^{HYBRID}\left(\alpha ,k\right)=\left\{\begin{array}{cc} \frac{\sigma_{i}^{2}+\alpha }{\sigma_{i}^{2}}, & \mathrm{if}\;i\leq k;\\ 0, & \mathrm{otherwise}; \end{array}\right.$$

with k∈{1,…,N} and $\alpha \in R^{+}$. By properly choosing the parameters *k* and α, the filter factors for low-frequency components can be set close to one while filter factors for high-frequency components can be set close to zero. Figure 1 depicts the behaviour of the filter factors obtained for the value $\alpha =2.5\cdot 10^{5}$ versus the singular values $\sigma_{i}$. The $\sigma_{i}$ plotted on the abscissa axes are the singular values of the matrix K of the experiment with simulated NMR data (see Section 4.3). We can observe that for singular values $\sigma_{i}$ larger than $\sqrt{\alpha }=500$ the filter factors $\phi_{i}^{HYBRID}$ behave as $\phi_{i}^{TIKH}$ (black dashdotted line) while for smaller values they are as $\phi_{i}^{TSVD}$ (red dashed line).

We observe that also the VSH solution can be expressed in terms of the SVD of K (see algorithm details in Appendix C). We define the index subset $I(k_{1},k_{2})$ including the indices of the singular values of K which correspond to singular values of $K_{k_{2},2}⊗K_{k_{1},1}$:

$$I\left(k_{1},k_{2}\right)=\left\{i\;|\;\sigma_{i}\in \mathrm{diag}\left({\bar{\Sigma }}_{k_{2},2}⊗{\bar{\Sigma }}_{k_{1},1}\right)\right\}.$$

Thus, we have:

(31) $$f_{VSH}=\sum_{i\in I(k_{1},k_{2})}\phi_{i}^{VSH}\left(\alpha ,k_{1},k_{2}\right)\frac{u_{i}^{T}s}{\sigma_{i}^{2}}{\hat{v}}_{i},\;{\hat{v}}_{i}=D_{VSH}^{*}v_{i}$$

where $D_{VSH}^{*}$ is the diagonal matrix (20) defined with respect to the active set $A^{*}$ of a local solution of (6) and the filter factors are

(32) $$\phi_{i}^{VSH}\left(\alpha ,k_{1},k_{2}\right)=\left\{\begin{array}{cc} \frac{\sigma_{i}^{2}+\alpha }{\sigma_{i}^{2}}, & i\in I(k_{1},k_{2});\\ 0, & \mathrm{otherwise}; \end{array}\right.$$

with $k_{1}\in \left\{1,\ldots ,N_{1}\right\}$, $k_{2}\in \left\{1,\ldots ,N_{2}\right\}$ and $\alpha \in R^{+}$. However, it is almost impossible to determine values of the truncation parameters $k_{1}$ and $k_{2}$ such that the vectors ${\hat{v}}_{i}$ for $i\in I(k_{1},k_{2})$ only correspond to low-frequency components including meaningful information about the unknown solution. An explanatory example will be further discussed in the numerical Section 4. For this reason, the VSH method cannot be considered a low-pass filtering method.

## 4. Numerical Results

In this section, we present some results obtained by our numerical tests with both simulated and real 2DNMR measurements. We have compared the proposed Hybrid method with the VSH method which is a reference method for 2DNMR data inversion. Moreover, in the case of real data, the UPEN method has been considered as a benchmark method. The considered methods have been implemented in Matlab R2018b on Windows 10 (64-bit edition), configured with Intel Core i5 3470 CPU running at 3.2GHz.

The relative error value, computed as $∥F_{ex}-F∥/∥F_{ex}∥$, is used to measure the effectiveness of the compared methods, while the execution time in seconds is used to evaluate their efficiency (here, $F_{ex}$ and F respectively denote the exact and restored distributions). The reported values of the execution time are the mean over ten runs. Since both methods are iterative, in the following, we give some details about the implemented stopping criteria and parameters.

### 4.1. Hybrid Method

The NP method is used for the solution of problem (11); it is described in detail in the Appendix B. As initial iterate the constant distribution with values equal to one is chosen. The NP iterations have been stopped on the basis of the relative decrease in the objective function F(f) of (11); i.e., the method is arrested when an iterate $f^{\left(k\right)}$ has been determined such that

(33) $$\frac{F\left(f^{\left(k\right)}\right)-F\left(f^{\left(k-1\right)}\right)}{F\left(f^{\left(k\right)}\right)}<{\mathrm{Tol}}_{\mathrm{NP}}.$$

The inner linear system for the search direction computation is solved by the CG method with relative tolerance ${\mathrm{Tol}}_{\mathrm{CG}}$. The values ${\mathrm{Tol}}_{\mathrm{NP}}=10^{-3}$ and ${\mathrm{Tol}}_{\mathrm{CG}}=10^{-7}$ have been fixed in all the numerical experiments. A maximum of ${\mathrm{Kmax}}_{\mathrm{NP}}=500$ and ${\mathrm{Kmax}}_{\mathrm{CG}}=10^{4}$ iterations have been allowed for NP and CG, respectively.

### 4.2. VSH Method

The VSH method consists of three main steps. In the first step, the data is compressed using SVDs of the kernels; in the second step, the constrained optimization problem is transformed to an unconstrained one in a lower dimensional space, by using a method adapted from the BRD algorithm. In the third step, the optimal value of α is selected by iteratively finding a solution with fit error similar to the known noise variance. The VSH method is described in detail in the Appendix C. Here we just report the values of the parameters required in our Matlab implementation of the VSH method.

Each iteration of the VSH method needs, for a fixed value of α, the solution of a reduced-size optimization problem obtained from (6) by projecting the data s onto the column subspace of $K_{k_{1},k_{2}}$. The Newton method is used for the subproblems solution; its iterations have been stopped on the basis of the relative decrease in the objective function. The values ${\mathrm{Tol}}_{N}=10^{-3}$ and ${\mathrm{Kmax}}_{N}=500$ have been fixed. The values ${\mathrm{Tol}}_{\mathrm{CG}}=10^{-7}$ and ${\mathrm{Kmax}}_{\mathrm{CG}}=10^{4}$ have been set for the CG method solving the inner linear system. The outer iterations of VSH have been terminated when two sufficiently close approximations of the unknown distribution have been determined or after a maximum of 100 iterations; the relative tolerance value ${\mathrm{Tol}}_{\mathrm{VSH}}=0.1$ has been set. As initial choice for the regularization parameter, the value $\alpha_{0}=1$ has been chosen.

### 4.3. Simulated NMR Data

**Figure 2:**
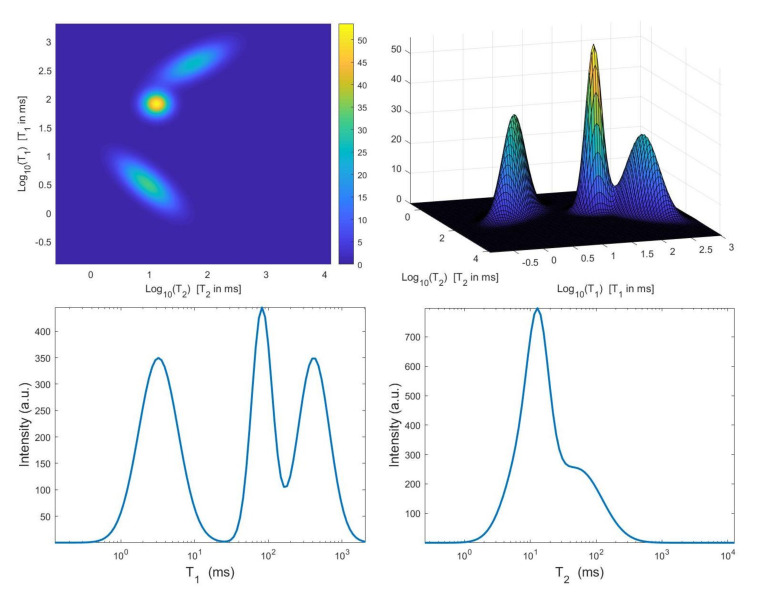
**Top**: simulated $T_{1}$–$T_{2}$ map and surface distribution. **Bottom**: projections along the $T_{1}$ and $T_{2}$ dimensions.

The considered simulated distribution $F(T_{1},T_{2})$, shown in Figure 2, is a mixture of three Gaussian functions located at $\left(T_{1},T_{2}\right)$ given by (81.86,12.84), (8.59,6.66) and (433.27,59.4) ms with standard deviations (0.1,0.1), (0.1,0.25) and (0.25,0.1) ms. We have considered the Laplace-type kernels $K_{1}$ and $K_{2}$ of (2), we have sampled $t_{1}$ logarithmically between 0.001 and 3, and $t_{2}$ linearly between 0.001 and 1 with $N_{1}=100$, $N_{2}=100$. The 2D data have been obtained according to the 2D observation model (3) with $M_{1}=2048$, $M_{2}=128$. A white Gaussian noise has been added to get a signal-to-noise ratio (SNR) equal to 23 dB. We remark that this environmental setting corresponds to a realistic $T_{1}$–$T_{2}$ measurement.

#### 4.3.1. Models Comparison

**Figure 3:**
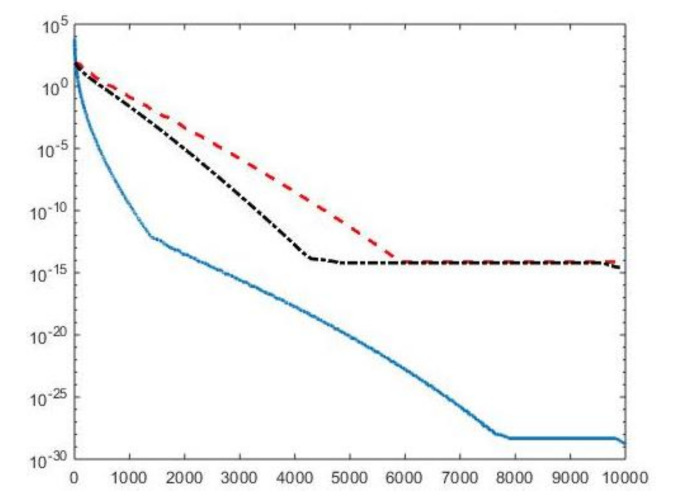
The singular values of K (blue line), $K_{1}$ (red dashed line) and $K_{2}$ (black dashdotted line). For easier visualization, the singular values of $K_{1}$ and $K_{2}$ are plotted versus the integers $1,100^{1},100^{2},\ldots ,100^{100}$.

**Figure 4:**
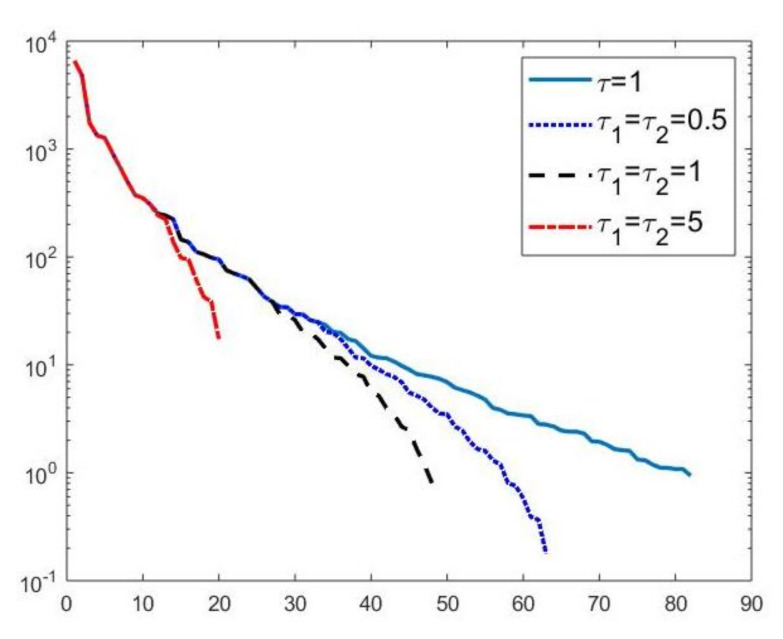
Singular values of $K_{k}$ obtained for τ=0.1 (blue line) and the singular values of $K_{k_{1},1}⊗K_{k_{2},2}$ for $\tau_{1}=\tau_{2}=0.5$ (blue dotted line), $\tau_{1}=\tau_{2}=1$ (black dashed line) and for $\tau_{1}=\tau_{2}=5$ (red dashdotted line).

We first compare the proposed hybrid inversion model (9) with the VSH model (6) and with classic Tikhonov model (4). Figure 3 depicts the singular values of K, $K_{1}$ and $K_{2}$. Clearly, the singular values of K are obtained by reordering in non increasing order the diagonal elements of $\Sigma =\Sigma_{2}⊗\Sigma_{1}$ and they are different from the diagonal elements of $\Sigma_{k_{1},1}⊗\Sigma_{k_{2},1}$. That is, some unwanted small singular values of K may be included in $K_{k_{1},1}⊗K_{k_{2},2}$, while some large singular values may be not considered. Figure 4 shows the singular values of the $K_{k}$ obtained for τ=1 (blue line) and the singular values of $K_{k_{1},1}⊗K_{k_{2},2}$ for $\tau_{1}=\tau_{2}=0.5$ (blue dotted line), $\tau_{1}=\tau_{2}=1$ (black dashed line) and for $\tau_{1}=\tau_{2}=5$ (red dashdotted line). Observe that the singular values of $K_{k}$ are always different from those of $K_{k_{1},1}⊗K_{k_{2},2}$. Considering the threshold value $\tau =\tau_{1}=\tau_{2}=1$, we have that the number k=82 of singular values of $K_{k}$ is larger than that of $K_{k_{1},k_{2}}$, given by $k_{1}\times k_{2}=8\times 6$. Comparing, in Figure 4, the blue line and the black dashed one (obtained for $\tau_{1}=\tau_{2}=1$), we observe that the singular values of $K_{k_{1},k_{2}}$ include a few elements smaller than τ, and miss some larger terms that should be included. Moreover, if $\tau_{1}=\tau_{2}=0.5$, we have that the number of singular values of $K_{k_{1},k_{2}}$ is $k_{1}\times k_{2}=9\times 7$ which is closer to k=82 but there are many singular values smaller than τ. Finally, in the case $\tau_{1}=\tau_{2}=5$, $K_{k_{1},k_{2}}$ has only a few large singular values ($k_{1}=5,k_{2}=4$) and many relevant solution coefficients are missing. The plots in Figure 4 indicate that, when considering problem (6), it is highly probable to include in the solution also those components dominated by noise (if τ is slightly too small) or to discard those components dominated by the contributions from the exact right-hand side (if τ is too large).

**Figure 5:**
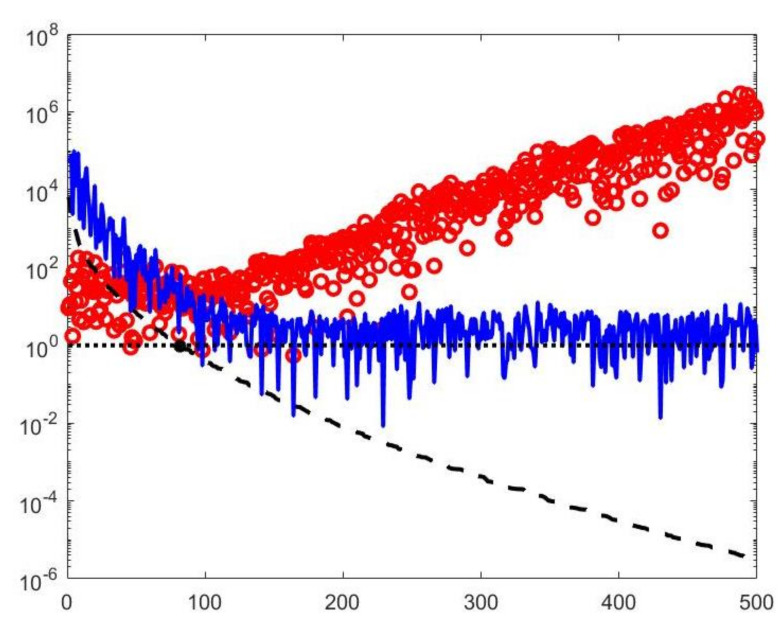
The Picard plot for the simulated NMR data. Singular values $\sigma_{i}$ (black dashed line), Fourier coefficients $u_{i}^{T}s$ (blue continuous line) and solution coefficients $u_{i}^{T}s/\sigma_{i}$ (red circles) for i=1,…,500.

A visual inspection of the Picard plot (Figure 5) indicates, for the hybrid method, the choice τ=1 for the truncation tolerance giving the value k=82 of the truncation parameter. The previous considerations suggest to choose the values $\tau_{1}=\tau_{2}=1$ for the VSH method.

Once defined the projection subspaces for VSH and Hybrid methods, we solve the optimization problems (6) and (11) by applying the same numerical solver (Newton Projection method NP) and changing the values of the regularization parameters α. In this way we want to investigate how the different subspace selections affect the solutions computed by the (6) and (11) models for different values of α. Moreover, we apply the Tikhonov method (4) in which no subspace projection is applied. For this reason, we use NP to solve all the different optimization problems, since we aim at comparing the three different models for data inversion, independently from the numerical method used for their solution.

**Figure 6:**
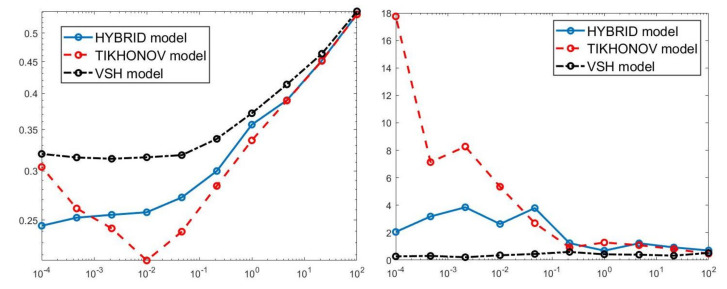
Relative error behaviour (**left**) and the computational time in seconds (**right**) versus the regularization parameter values.

**Figure 7:**
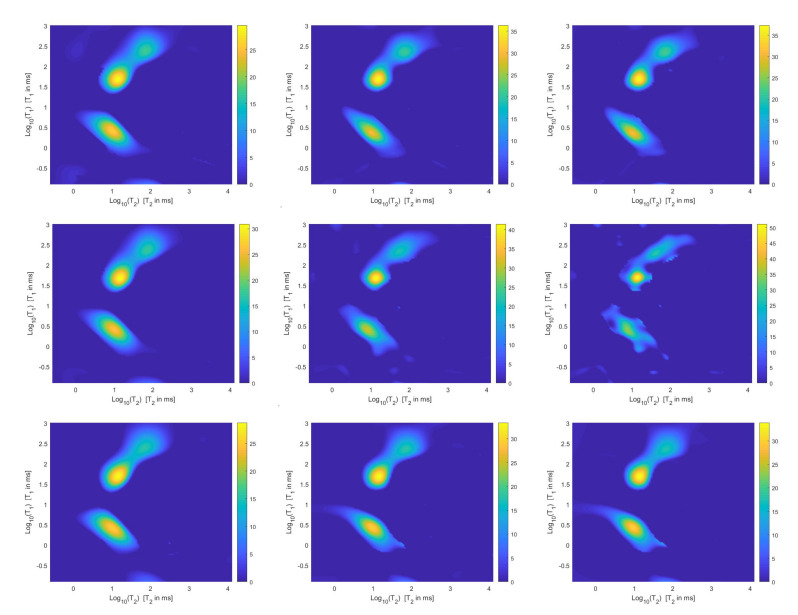
Estimated distributions from the Hybrid (**top row**), Tikhonov (**middle row**) and VSH (**bottom row**) models for α=1 (**left column**), $\alpha =10^{-2}$ (**middle column**) and $\alpha =10^{-4}$ (**right column**).

**Figure 8:**
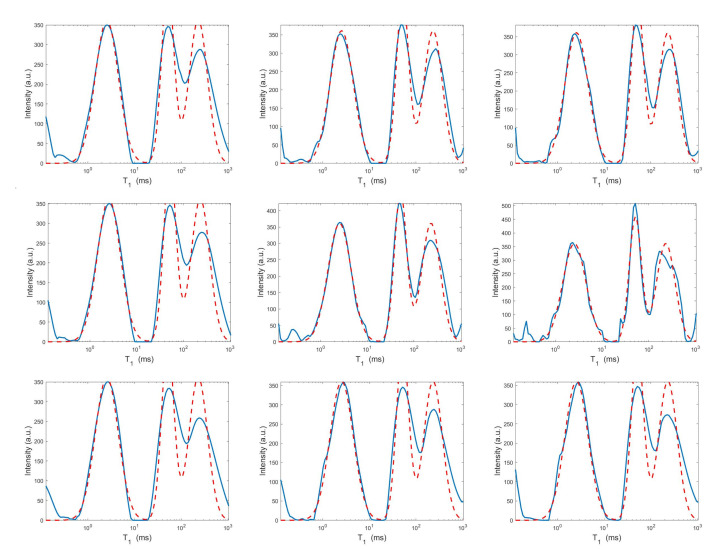
Projections along the T1 dimension from the Hybrid (**top row**), Tikhonov (**middle row**) and VSH (**bottom row**) models for α=1 (**left column**), $\alpha =10^{-2}$ (**middle column**) and $\alpha =10^{-4}$ (**right column**). The red dashed and blue continuous lines respectively represent the exact projections and the computed ones.

**Figure 9:**
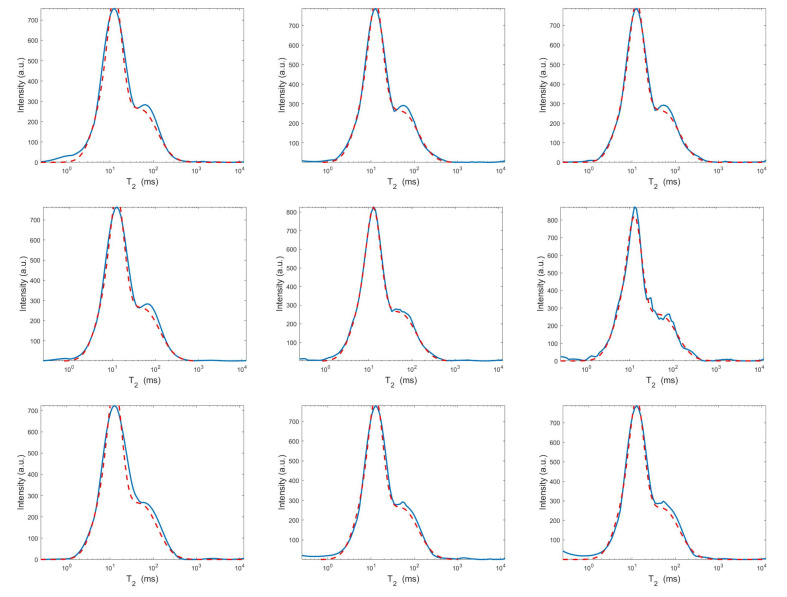
Projections along the T2 dimension from the Hybrid (**top row**), Tikhonov (**middle row**) and VSH (**bottom row**) models for α=1 (**left column**), $\alpha =10^{-2}$ (**middle column**) and $\alpha =10^{-4}$ (**right column**). The red dashed and blue continuous lines respectively represent the exact projections and the computed ones.

For each model and for ten values of α, logarithmically distributed between $10^{-4}$ and $10^{2}$, Table 1 reports the relative error values (columns labeled *Err*), the number of NP iterations (columns labeled *It*), and the time in seconds required for solving the three inversion models (columns labeled *Time*). The numerical results of Table 1 are summarized in Figure 6 where the relative error behaviour (on the left) and the computational time in seconds (on the right) are shown versus the regularization parameter values. Figure 7 shows the distributions estimated from the three models, for α=1 (left column), $\alpha =10^{-2}$ (middle column) and $\alpha =10^{-4}$ (right column). Figure 8 and Figure 9 report the corresponding projections along the $T_{1}$ and $T_{2}$ dimensions. Figure 7, Figure 8 and Figure 9 shows the importance of the proper choice of the subspace where the computed solution is represented. Indeed, the distribution computed by our method lies in a subspace of the column space of $V_{k}$ while the distribution determined by the VSH method belongs to a subspace of the space spanned by the column vectors of $V_{k_{2},2}⊗V_{k_{1},1}$.

The results of Table 1 and the plot of Figure 6 and Figure 7 show that the Hybrid model (9) is less sensitive to small values of α than the Tikhonov one, because the matrix $K_{k}$ is better conditioned than K. When the value of α is properly chosen the quality of distributions given by these two models is comparable, but the solution of (9) requires less computational effort. Also the VSH model is less sensitive to small values of α, because $K_{k_{1},k_{2}}$ is better conditioned than K. Its solution has the least computational cost but the computed distributions exhibit artifacts which are due to subspace where they are represented (see Figure 7, Figure 8 and Figure 9).

#### 4.3.2. Methods Comparison

**Figure 10:**
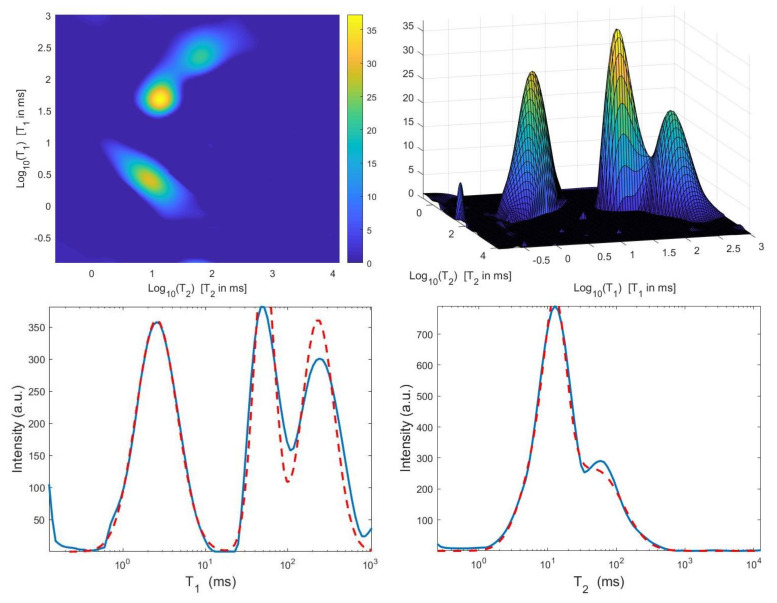
Hybrid method: restored $T_{1}$–$T_{2}$ distribution and projections along the T1 and T2 dimensions (τ=0.05).

**Figure 11:**
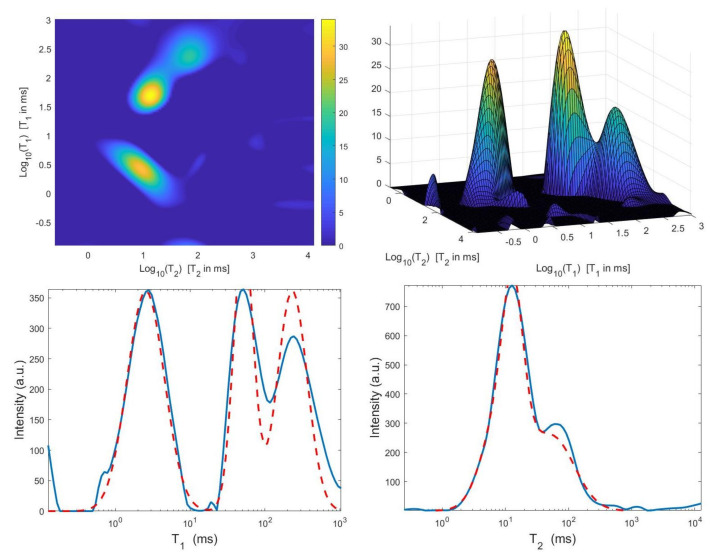
VSH method: restored $T_{1}$–$T_{2}$ distribution and projections along the T1 and T2 dimensions (τ=0.1).

We now compare the Hybrid and VSH algorithms on the simulated NMR data. Table 2 reports the relative error values and the required time in seconds for several values of the truncation parameter τ (Here, $\tau_{1}=\tau_{2}=\tau$). Moreover, the table shows the computed value for the regularization parameter α, the number of performed Newton (or Newton Projection) and CG iterations (columns labeled *It N* and *It CG*, respectively) and it reports, only for VSH, the number of outer iterations (column labeled *It*). The numerical results show that the VSH method has the lowest computational complexity while the Hybrid method gives the most accurate distributions. The execution time of the Hybrid method is very low, although VSH is less time consuming. Figure 10 and Figure 11 depict the best distributions estimated by the Hybrid and VSH methods, i.e.,: the distribution obtained with τ=0.05 and $\tau_{1}=\tau_{2}=0.1$, respectively. By visually comparing Figure 10 and Figure 11, we observe some spurious small oscillations in the VSH distribution both in the boundary and in the flat region, while the distribution computed by the Hybrid method is less affected by such artefacts.

### 4.4. Real NMR data

In this section we compare the Hybrid and VSH methods using real MR measurements obtained from the analysis of an egg yolk sample. The sample was prepared in NMR Laboratory at the Department of Physics and Astronomy of the University of Bologna, by filling a 10 mm external diameter glass NMR tube with 6 mm of egg yolk. The tube was sealed with Parafilm, and then at once measured. NMR measurements were performed at 25 °C by a homebuilt relaxometer based on a PC-NMR portable NMR console (Stelar, Mede, Italy) and a 0.47 T Joel electromagnet. All relaxation experimental curves were acquired using phase-cycling procedures. The π/2 pulse width was of 3.8µs and the relaxation delay (RD) was set to a value greater than 4 times the maximum $T_{1}$ of the sample. In all experiments RD was equal to 3.5 s. For the 2D measurements, longitudinal-transverse relaxation curve ($T_{1}$-$T_{2}$) was acquired by an IR-CPMG pulse sequence (RD-$\pi_{x}$-$T_{I}$-${\left(\pi /2\right)}_{x}$-TE/2-[$\pi_{y}$-TE/2-echo acquisition-TE/2]${}_{\mathrm{NE}}$). The $T_{1}$ relaxation signal was acquired with 128 inversion times ($T_{I}$) chosen in geometrical progression from 1 ms up to 2.8 s, with NE=1024 (number of acquired echos, echo times TE=500µs) on each CPMG, and number of scans equal to 4. All curves were acquired using phase-cycling procedures.

The data acquisition step produces an ascii structured file (in the STELAR data format) including $M_{1}\cdot M_{2}$ relaxation data s in (3) where $M_{1}=128$ and $M_{2}=1024$, and the vectors $t_{1}\in R^{M_{1}}$($T_{I}$ inversion times), $t_{2}\in R^{M_{2}}$(CPMG echo times). The file is freely available upon email request to the authors. For the data inversion, in order to respect approximately the same ratio existing between $M_{1}$ and $M_{2}$, we choose the values $N_{1}=64$, $N_{2}=73$ and compute the vectors $T_{1}$, $T_{2}$ in geometric progression in the ranges of predefined intervals obtained from the minimum and maximum values of the vectors $t_{1},t_{2}$ respectively. Finally, using (2) we compute the matrices $K_{1}$ and $K_{2}$.

**Figure 12:**
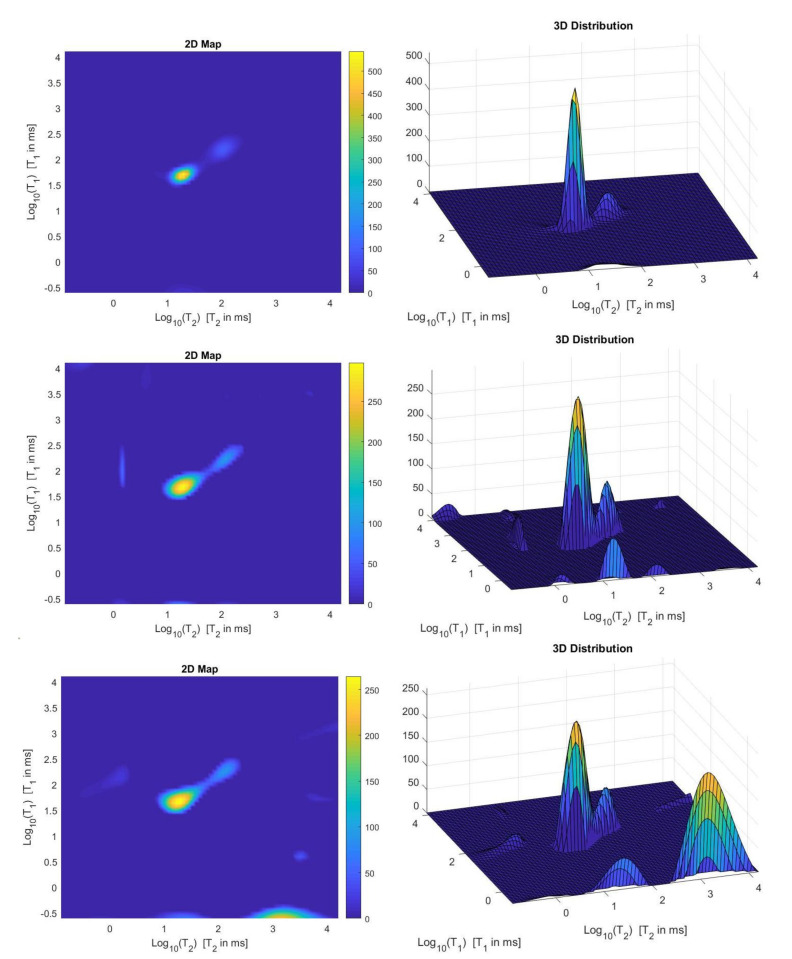
From **top** to **bottom**: UPEN, Hybrid and VSH restored $T_{1}$–$T_{2}$ maps (**left**) and surface distributions (**right**).

We use the times distribution restored by the 2DUPEN method [14], shown in Figure 12 (top line), as benchmark distribution as the UPEN method uses multiparameter regularization and it is known to provide accurate results [3]. Obviously, 2DUPEN requires more computational effort since it involves the estimation of one regularization parameter for each pixel of the distribution.

**Figure 13:**
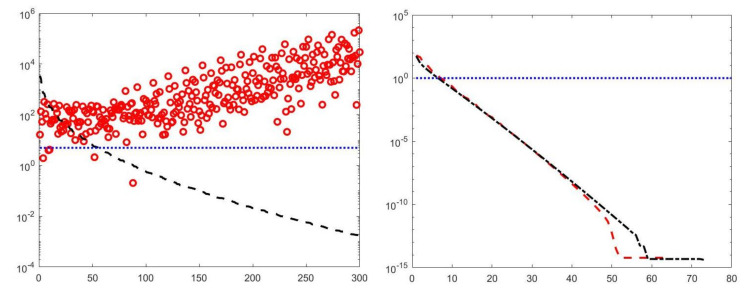
**Left**: singular values (black dashed line) of K and solution coefficients (red circles). Only the first 300 singular values are depicted. **Right**: Singular values of $K_{1}$ (red dashed line) and $K_{2}$ (black dashdotted line). The horizontal line corresponds to the value τ=1.

**Figure 14:**
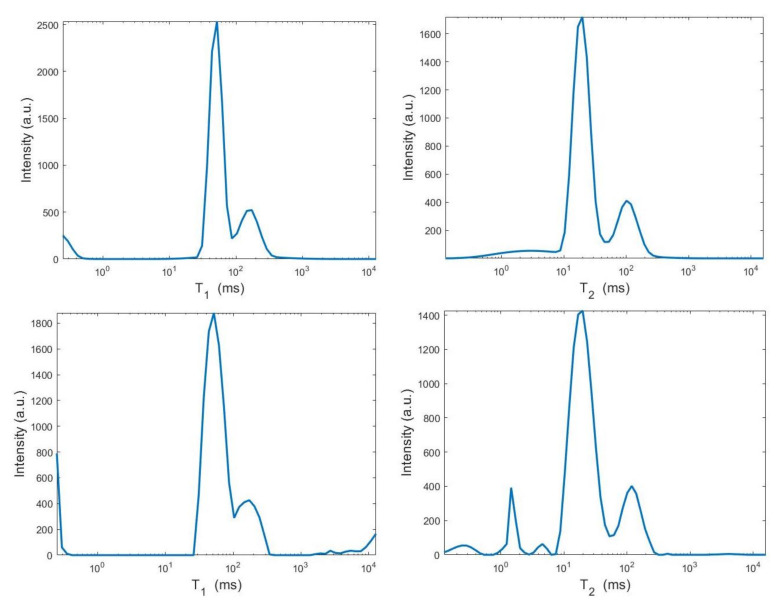

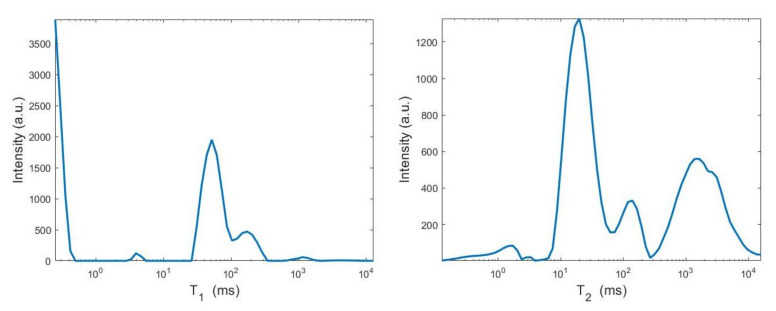
From **top** to **bottom**: UPEN, Hybrid and VSH projections along the T1 (**left**) and T2 (**right**) dimensions.

By visual inspection of the Picard plot (Figure 13) the value τ=1 has been chosen for the hybrid method; the same value is fixed for the VSH method. Figure 13 shows the singular values of K, $K_{1}$ and $K_{2}$. For the VSH method, we report the results obtained at the first iteration since, in this case, they worsen as the iteration number increases. Table 3 reports the $T_{2}-T_{1}$ coordinates (in ms) where a peak is located, its hight in a.u. (arbitrary unit) and the required computational time in seconds. Finally, Figure 12 and Figure 14 illustrate the distribution maps, the surfaces restored and the projections along the T1 and T2 dimensions; the results of the Hybrid method are more similar to those obtained by 2DUPEN, while the distribution provided by the VSH method seems to exhibits larger boundary artifacts.

## 5. Conclusions

In this paper, we have presented a hybrid approach to the inversion of 2DNMR measurements. This approach combines main features of Tikhonov and TSVD regularization in order to jointly select a suitable approximation subspace for the restored distribution and to reduce the computational cost of the inversion. The Picard condition is used to select, at the same time, the Tikhonov regularization parameter and the SVD truncation parameter. The numerical results show that the proposed hybrid method is effective, efficient and robust. In our future work, we intend to complement the presented hybrid approach in the 2DUPEN method.

## Figures and Tables

**Table 1:** Numerical results for the models comparison on simulated NMR data. Results obtained by applying NP method to (6), (4) and (11) with $\tau =\tau_{1}=\tau_{2}=1$.

	Hybrid			Tikh			VSH		
α	*Err*	*Time*	*It*	*Err*	*Time*	*It*	*Err*	*Time*	*It*
100	0.536	0.90	25	0.536	0.65	20	0.542	0.73	52
21.54	0.452	1.29	31	0.451	1.11	25	0.463	0.52	33
4.6416	0.390	1.74	31	0.389	1.56	26	0.413	0.65	33
1	0.356	0.99	15	0.336	1.79	26	0.372	0.70	36
0.2154	0.300	1.57	18	0.284	1.29	15	0.338	0.97	39
0.0464	0.272	5.39	47	0.240	3.75	33	0.318	0.73	27
0.01	0.257	3.87	31	0.215	7.43	53	0.316	0.58	23
0.0022	0.255	5.55	43	0.243	11.41	68	0.314	0.35	15
0.0005	0.252	4.73	32	0.261	9.94	48	0.315	0.51	20
0.0001	0.245	2.94	25	0.304	24.91	100	0.320	0.45	18

**Table 2:** Numerical results for the methods comparison on simulated NMR data.

τ	*Method*	α	*Err*	*Time*	*It*	*It N*	*It CG*
0.001	Hybrid	$1.73\;10^{-6}$	$2.35\;10^{-1}$	0.68	/	18	1700
	VSH	$1.06\;10^{-1}$	$3.08\;10^{-1}$	1.60	50	198	14,800
0.005	Hybrid	$6.52\;10^{-5}$	$2.34\;10^{-1}$	0.75	/	21	2000
	VSH	$9.34\;10^{-2}$	$3.85\;10^{-1}$	1.50	50	198	14,800
0.01	Hybrid	$1.66\;10^{-4}$	$2.32\;10^{-1}$	0.62	/	18	1700
	VSH	$9.34\;10^{-2}$	$3.85\;10^{-1}$	1.50	50	198	14,800
0.05	Hybrid	$6.80\;10^{-3}$	$2.44\;10^{-1}$	0.45	/	13	2200
	VSH	$8.52\;10^{-2}$	$3.15\;10^{-1}$	0.65	28	112	8400
0.1	Hybrid	$1.35\;10^{-2}$	$2.41\;10^{-1}$	0.80	/	23	2200
	VSH	$7.96\;10^{-2}$	$3.02\;10^{-1}$	0.11	4	20	1600
0.5	Hybrid	$6.15\;10^{-1}$	$3.23\;10^{-1}$	0.95	/	21	2000
	VSH	$3.93\;10^{-2}$	$3.12\;10^{-1}$	0.19	6	35	2900
1	Hybrid	$1.17\;10^{0}$	$3.23\;10^{-1}$	0.79	/	23	2200
	VSH	$5.07\;10^{-2}$	$3.43\;10^{-1}$	0.25	8	53	4500
5	Hybrid	$5.51\;10^{+1}$	$5.08\;10^{-1}$	1.36	/	43	3779
	VSH	$9.26\;10^{-2}$	$6.80\;10^{-1}$	0.06	2	27	1008
10	Hybrid	$1.16\;10^{+2}$	$5.08\;10^{-1}$	0.53	/	20	1496
	VSH	$5.99\;10^{-2}$	$7.03\;10^{-1}$	0.10	6	50	1153

**Table 3:** Location and height of the peak in the restored distribution and required execution time in seconds.

*Method*	T2	T1	*Peak Height*	*Time*
Hybrid	19.85	51.90	269.52	11.06
VSH	19.85	43.70	264.48	1.24
UPEN	19.85	51.90	544.23	71.93

## Data Availability

No new data were created or analyzed in this study. Data sharing is not applicable to this article.

## Appendix A

**Proof.** (Lemma 1) The thesis is obtained if we verify that $D^{*}V\Sigma^{†}U^{T}\in R^{N\times M}$ meets the four Moore-Penrose conditions which define the pseudo-inverse $A^{†}$ of A as the unique matrix satisfying

1. $AA^{†}A=A$;
2. $A^{†}AA^{†}=A^{†}$;
3. ${\left(AA^{†}\right)}^{H}=AA^{†}$;
4. ${\left(A^{†}A\right)}^{H}=A^{†}A$.

By substituting $U\Sigma V^{T}D^{*}$ for A and $D^{*}V\Sigma^{†}U$ for $A^{†}$ in the first condition, we have:

$$U\Sigma V^{T}D^{*}\left(D^{*}V\Sigma^{†}U^{T}\right)U\Sigma V^{T}D^{*}=U\Sigma V^{T}D^{*}(D^{*}\left(V\left(\Sigma^{†}\left(\left(U^{T}U\right)\Sigma \right)V^{T}\right)D^{*}\right)=U\Sigma V^{T}D^{*}$$

since $\Sigma^{†}\Sigma =I_{N}$, $U^{T}U=I_{M}$, $VV^{T}=I_{N}$ and $D^{*}D^{*}=D^{*}$. Analogously, we can prove the second condition:

(A1) $$D^{*}V\Sigma^{†}U^{T}\left(U\Sigma V^{T}D^{*}\right)D^{*}V\Sigma^{†}U^{T}=D^{*}V\Sigma^{†}U.$$

We have that

(A2) $${\left(AA^{†}\right)}^{H}={\left(U\Sigma V^{T}D^{*}D^{*}V\Sigma^{†}U^{T}\right)}^{H}=U\Sigma^{†}V^{T}D^{*}D^{*}V\Sigma U^{T}=AA^{†}.$$

The last condition follows analogously. □

## Appendix B. The Newton Projection Method

Let us now denote by $Q^{\left(k\right)}\left(f\right)$ the objective function of (11):

(A3) $$Q^{\left(k\right)}\left(f\right)=∥\Sigma_{k}V_{k}^{T}f-U_{k}^{T}{s∥}^{2}+\alpha {∥f∥}^{2}$$

and by A(f) the set of indices defined as [24]

$$A\left(f\right)=\left\{i\;|\;0\leq f_{i}\leq \epsilon \;\mathrm{and}\;{\left(\nabla Q\right)}_{i}>0\right\},\;\epsilon =\min\{\bar{\epsilon }{,∥f-\left[f-\nabla Q\right]}^{+}∥\}$$

where $\bar{\epsilon }$ is a small positive parameter and ${\left[\cdot \right]}^{+}$ denotes the projection on the positive orthant. Finally, let E and F denote the diagonal matrices [24] such that

$$\begin{array}{cc} {\left\{E\left(f\right)\right\}}_{ii} & =\left\{\begin{array}{cc} 1, & i\notin A(f);\\ 0, & i\in A(f); \end{array}\right.\\ F(f) & =I-E(f). \end{array}$$

The NPCG method for the minimization of $Q^{\left(k\right)}\left(f\right)$ under nonnegativity constraints is formally stated in Algorithm A1.

**Algorithm A1:** NP (Newton-Projection) algorithm
1: choose $f^{\left(0\right)}$ and set k=0
2: **for** k=1,2,… **do**
3: compute the index subset $A^{\left(k\right)}$ and the matrices $E^{\left(k\right)}$ and $F^{\left(k\right)}$;
4: etermine the search direction $d^{\left(k\right)}$ by solving, with the CG method, the linear system $$\left(E^{\left(k\right)}\nabla^{2}Q^{\left(k\right)}\left(f^{\left(k\right)}\right)E^{\left(k\right)}+F^{\left(k\right)}\right)d=-\nabla Q^{\left(k\right)}\left(f^{\left(k\right)}\right);$$
5: determine a step-length $\alpha^{\left(k\right)}$ satisfying the Armijo rule along the projection arc [23];
6: compute $f^{\left(k+1\right)}={\left[f^{\left(k\right)}+\alpha^{\left(k\right)}d^{\left(k\right)}\right]}^{+}$;
7: **end for**

## Appendix C. The Venkataramanan-Song-Hürlimann Algorithm

Let $K_{i}={\bar{U}}_{i}{\bar{\Sigma }}_{i}{\bar{V}}_{i}^{T}$ be the SVD of $K_{i}$, i=1,2 and let $k_{i}$ be the number of considered singular values; for example, $k_{i}$ can be the last index of the singular values than a given tolerance τ, i=1,2. Given the TSVD matrices $K_{k_{1},1}={\bar{U}}_{k_{1},1}{\bar{\Sigma }}_{k_{1},1}{\bar{V}}_{k_{1},1}^{T}$ and $K_{k_{2},2}={\bar{U}}_{k_{2},2}{\bar{\Sigma }}_{k_{2},2}{\bar{V}}_{k_{2},2}^{T}$ of $K_{1}$ and $K_{2}$, and defined their Kroenecker product $K_{k_{1},k_{2}}$:

(A4) $$K_{k_{1},k_{2}}=K_{k_{2},2}⊗K_{k_{1},1}$$

problem (4) can be approximated as

(A5) $$\min_{f\geq 0}\;∥K_{k_{1},k_{2}}{f-s∥}^{2}+\alpha {∥f∥}^{2}$$

or equivalently

(A6) $$\min_{F\geq 0}\;∥K_{k_{1},1}FK_{k_{2},2}^{T}{-S∥}^{2}+\alpha {∥F∥}^{2}.$$

The first step of the VSH algorithm consists of projecting the data s onto the column subspace of $K_{k_{1},k_{2}}$:

$$P_{1,2}\left(s\right)=\left({\bar{U}}_{k_{1},1}⊗{\bar{U}}_{k_{2},2}\right){\left({\bar{U}}_{k_{1},1}⊗{\bar{U}}_{k_{2},2}\right)}^{T}s$$

that is

$$P_{1,2}\left(S\right)={\bar{U}}_{k_{1},1}{\bar{U}}_{k_{1},1}^{T}S{\bar{U}}_{k_{2},2}{\bar{U}}_{k_{2},2}^{T}.$$

Hence, by neglecting constant terms, problem (A6) can now be equivalently rewritten as

(A7) $$\min_{F\geq 0}\;∥{\bar{U}}_{k_{1},1}{\bar{U}}_{k_{1},1}^{T}K_{k_{1}}FK_{k_{2}}^{T}{\bar{U}}_{k_{2},2}{\bar{U}}_{k_{2},2}^{T}-P_{1,2}{\left(S\right)∥}^{2}+\alpha {∥F∥}^{2}$$

which becomes

(A8) $$\min_{F\geq 0}\;∥\left({\bar{\Sigma }}_{k_{1},1}{\bar{V}}_{k_{1},1}^{T}\right)F{\left({\bar{\Sigma }}_{k_{2},2}{\bar{V}}_{k_{2},2}^{T}\right)}^{T}-{\bar{U}}_{k_{1},1}^{T}S{\bar{U}}_{k_{2},2}{∥}^{2}+\alpha {∥F∥}^{2}$$

or, equivalently

(A9) $$\min_{f\geq 0}\;∥\tilde{K}f-\tilde{s}{∥}^{2}+\alpha {∥f∥}^{2}$$

where

(A10) $$\tilde{K}={\bar{\Sigma }}_{k_{2},2}{\bar{V}}_{k_{2},2}^{T}⊗{\bar{\Sigma }}_{k_{1},1}{\bar{V}}_{k_{1},1}^{T}\;\mathrm{and}\;\tilde{s}=\left({\bar{U}}_{k_{2},2}^{T}⊗{\bar{U}}_{k_{1},1}^{T}\right)s.$$

This results in compressed data $\tilde{s}$ and kernels ${\bar{\Sigma }}_{k_{1},1}{\bar{V}}_{k_{1},1}^{T}$ and ${\bar{\Sigma }}_{k_{2},2}{\bar{V}}_{k_{2},2}^{T}$ of significantly smaller dimensions, thus avoiding large memory requirements and reducing computational effort.

The second step of VSH consists of solving the optimization problem (A9) for a specific value of α. In [4], this is done by using a method adapted from the Butler-Reeds-Dawson (BRD) algorithm [9] which maps the constrained optimization problem (A9) onto an unconstrained one. Let us define a vector c implicitly from f by

(A11) $$f=\max(0,{\tilde{K}}^{T}c).$$

Hence, problem (A9) can be reformulated as the unconstrained minimization problem

(A12) $$\min_{c}\;\left(\frac{1}{2}c^{T}\left(G(c)+\alpha I\right)c-c^{T}\tilde{s}\right),$$

where

$$G\left(c\right)=\tilde{K}\left(\begin{array}{cccc} H({\tilde{K}}_{1}^{T}c), & 0 & \ldots & 0\\ 0 & H({\tilde{K}}_{2}^{T}c) & \ldots & 0\\ \vdots & \vdots & \; & \vdots \\ 0 & 0 & \ldots & H({\tilde{K}}_{k_{1}k_{2}}^{T}c) \end{array}\right){\tilde{K}}^{T}$$

H(x) is the Heaviside function and I is the identity matrix. The Newton method is then used for solving problem (A12). The Conjugate Gradient (CG) method is employed for the solution of the inner linear system.

Once (A9) has been solved for a specific α, the BRD method is used for updating the value of α as

(A13) $$\alpha_{\mathrm{new}}=\frac{\sqrt{k_{1}k_{2}}}{∥c∥}.$$

This procedure is sketched in Algorithm A2.

**Algorithm A2:** VSH (Venkataramanan–Song–Hürlimann) algorithm
1: choose $k_{1}$ and $k_{2}$
2: compute the TSVD of $K_{1}$ and $K_{2}$
3: compute the kernel $\tilde{K}$ and the projected data $\tilde{s}$ as in (A10)
4: choose $\alpha^{\left(0\right)}$
5: **for** k=1,2,… **do**
6: compute with the Newton method a solution $c^{\left(j\right)}$ to the unconstrained problem $$\min_{c}\;\left(\frac{1}{2}c^{T}\left(G\left(c\right)+\alpha^{\left(j\right)}I\right)c-c^{T}\tilde{s}\right)$$
7: compute $f^{\left(j\right)}=\max\left(0,{\tilde{K}}^{T}c^{\left(j\right)}\right)$
8: update $\alpha^{\left(j+1\right)}=\sqrt{k_{1}k_{2}}/∥c^{\left(j\right)}∥$
9: **end for**
